# The need for a large-scale trial of fibrate therapy in diabetes: the rationale and design of the Fenofibrate Intervention and Event Lowering in Diabetes (FIELD) study. [ISRCTN64783481]

**DOI:** 10.1186/1475-2840-3-9

**Published:** 2004-12-01

**Authors:** The FIELD Study Investigators

**Affiliations:** 1 NHMRC Clinical Trials Centre, Mallett St Campus, University of Sydney NSW 2006, Australia

**Keywords:** diabetes mellitus, type 2, fibrate, cardiovascular disease, randomized controlled trial, coronary heart disease

## Abstract

**Background:**

Fibrates correct the typical lipid abnormalities of type 2 diabetes mellitus, yet no study, to date, has specifically set out to evaluate the role of fibrate therapy in preventing cardiovascular events in this setting.

**Methods:**

Subjects with type 2 diabetes, aged 50–75 years, were screened for eligibility to participate in a long-term trial of comicronized fenofibrate 200 mg daily compared with matching placebo to assess benefits of treatment on the occurrence of coronary and other vascular events. People with total cholesterol levels 3.0–6.5 mmol/L plus either a total-to-HDLc ratio >4.0 or triglyceride level >1.0 mmol/L with no clear indication for lipid-modifying therapy were eligible.

**Results:**

A total of 9795 people were randomized into the Fenofibrate Intervention and Event Lowering in Diabetes (FIELD) trial. All received dietary advice, followed by a 6-week single-blind placebo run-in, then a 6-week active run-in period before randomization. Participants are being followed up every 6 months for outcome events and safety assessments. The study is designed to yield at least 500 coronary events (primary endpoint: first nonfatal myocardial infarction or coronary death) over 5 years, to have 80% power to identify as statistically significant at 2*P *= 0.05 a 22% reduction in such events, using intention-to-treat methods.

**Conclusions:**

Type 2 diabetes is the most common endocrine disorder worldwide, and its prevalence is increasing. The current evidence about use of fibrates in type 2 diabetes, from around 2000 people treated, will increase with FIELD to evidence from around 12000. FIELD will establish the role of fenofibrate treatment in reducing cardiovascular risk in people with type 2 diabetes. The main results are expected to be available in late 2005.

## Introduction

Type 2 diabetes mellitus is an increasingly common condition associated with a high cardiovascular risk. To date, very few trials of lipid-lowering therapy have focused on this condition, and in particular, no large trials of fibrate therapy in diabetes have been conducted. As fibrates are known to correct the typical dyslipidaemia of diabetes, their role in cardiovascular risk reduction in diabetes may be especially important. The Fenofibrate Intervention and Event Lowering in Diabetes (FIELD) study is a multicentre, double-blind, placebo-controlled trial evaluating the effects on coronary morbidity and mortality of long-term treatment with fenofibrate to elevate high-density lipoprotein (HDL) cholesterol levels and lower triglyceride (TG) levels in patients with type 2 diabetes and total blood cholesterol between 3 and 6.5 mmol/L (115 and 250 mg/dL) at study entry. In type 2 diabetes, rates of coronary heart disease (CHD) are 3 to 4 times higher than those of persons without diabetes at any given level of blood cholesterol, and at any given age [1,2]. Evidence also suggests that in women with diabetes the natural protection against CHD afforded by sex may be lost [3,4]. Further, people with type 2 diabetes have both higher in-hospital mortality after myocardial infarction (MI) and a poorer outcome in the subsequent years [5,6], losing on average between 5 and 10 years of life expectancy. It follows that type 2 diabetes contributes significantly to the overall burden of premature CHD morbidity and mortality, far in excess of its prevalence in the community.

### Diabetes and blood lipids

Blood total cholesterol levels are not substantially different between patients with type 2 diabetes and those of nondiabetic populations of similar age and sex [7]. However, evaluation of other lipoprotein fractions shows that those with diabetes more often have a below-average HDL cholesterol level and elevation of TG levels in the blood [8,9], which together confer an independent additional risk of CHD [10,11]. Furthermore, although low-density lipoprotein (LDL) cholesterol levels are not substantially raised, the LDL particle is often smaller and denser than in similar nondiabetic populations, which is considered to be a more atherogenic state [12]. An increased number of LDL particles, as seen in diabetes, is reflected in an elevated level of plasma apolipoprotein B, a more powerful predictor of risk for cardiovascular events than either total cholesterol or LDL cholesterol [13].

The strength of the cholesterol-CHD relationship is very similar for those with type 2 diabetes as for nondiabetics, although at a higher background rate of CHD [2]. Evidence from the Helsinki Heart Study [14], which tested long-term fibrate (gemfibrozil) use in hypercholesterolaemic men and women without prior coronary disease, showed a significant reduction in coronary events, with the reduction among the small numbers of people with diabetes not being separately significant, but appearing somewhat greater [15]. The reductions in events observed were greater than would have been expected on the basis of lowering of LDL cholesterol alone. So, whether substantially increasing low HDL cholesterol levels and reducing elevated triglyceride levels independently reduces cardiovascular events and mortality and should be a specific target for therapy remains less well agreed.

### Why a large trial of fibrates?

For patients with type 2 diabetes and its typical dyslipidaemia, many physicians believe that fibrates are the logical first choice of drug treatment. The fibrates have been in clinical use for a long time, being well tolerated and with few short-term side-effects. Fenofibrate has been widely used and marketed for more than 20 years and is an effective agent for reducing plasma triglyceride and raising HDL cholesterol [16]. Although the effects on lipid fractions may vary with the population under study, a fall of 15% or more in total cholesterol, mediated through a reduction in LDL cholesterol, is often seen with long-term use [16]. In parallel, HDL cholesterol elevation of 10–15% is common, together with large reductions in plasma triglycerides of 30–40%. In addition, a reduction in plasma fibrinogen of about 15% has been observed [16].

FIELD is designed to provide the first properly randomized evidence as to whether the substantial effects of fenofibrate confer a benefit on clinical cardiovascular events in persons with type 2 diabetes. A clearly favourable result might be expected to help physicians determine which type of lipid-modifying drug therapy is likely to be most cost-effective for such people.

## The FIELD study design

FIELD is a randomized, double-blind, placebo-controlled parallel-group trial among middle-aged to elderly people with type 2 diabetes mellitus considered to be at increased risk of CHD. Those with and without pre-existing vascular disease or other lipid abnormalities, such as low HDL cholesterol and elevated TG, were eligible, provided the total blood cholesterol level at screening fell between 3.0 and 6.5 mmol/L (about 115–250 mg/dL) plus either a total-to-HDL cholesterol ratio of >4.0 or a blood TG level >1.0 mmol/L (88.6 mg/dL) (Table 1). The study is being conducted in 63 clinical centres in Australia (39), Finland (9) and New Zealand (15) (see Appendix).

**Table 1:** Inclusion and exclusion criteria for the FIELD study

Individuals were eligible for this study provided they had the following characteristics:
• male or female, aged 50–75 years inclusive
• non-insulin dependent diabetes mellitus (type 2) with age at diagnosis >35 years (currently using any of diet, tablets or insulin); for Maori, Pacific Islanders, Australian Aborigines and Torres Strait Islanders, the eligible age of diagnosis was >25 years, provided there had been at least 1 year of treatment without insulin
• on the basis of diabetes, considered to be at higher risk for coronary heart disease than the general population
• no clear indication for any cholesterol-lowering treatment: the patient was not already taking any cholesterol-lowering drug and neither the patient nor the patient's doctor considered there to be any definite need to do so
• total cholesterol level 3 to 6.5 mmol/L, plus either
a total cholesterol-to-HDL cholesterol ratio of ≥ 4.0
a blood triglyceride level >1.0 mmol/L
• no clear contraindication to study therapy in the view of the treating physician
• no other predominant medical problem that might limit compliance with 5 years of study treatment or compromise long-term participation and clinic attendance in the trial
Individuals were not eligible if they had any of the following characteristics:
• serum triglyceride >5 mmol/L in the baseline visit fasting blood sample
• concurrent treatment with any other lipid-lowering agent
• serum creatinine >130 μmol/L
• known chronic liver disease, transaminases >2 × upper limit of normal or symptomatic gall-bladder disease
• myocardial infarction or hospital admission for unstable angina within 3 months
• female, of child-bearing potential, unless sterilized or on reliable approved methods of contraception, including oral contraceptives
• concurrent cyclosporin treatment (or a condition likely to result in organ transplantation and the need for cyclosporin during the next 5 years)
• known allergy to any fibrate drug or known photosensitivity
• unwilling or unable to consent to enter the study, with the understanding that follow-up was planned to continue for more than 5 years

The underlying principle guiding recruitment of patients into the study was that of clinical uncertainty: that is, patients were only to be considered if the patients' treating physicians were substantially uncertain about the value of lipid-modifying therapy for that particular individual and felt that there was no indication for lipid-modifying therapy. Therefore, none of the participants was on lipid-lowering therapy at study entry.

Following clinical and laboratory screening for eligibility, informed consent, and completion of the run-in period, patients were randomized to receive either fenofibrate (200 mg comicronized formulation) or matching placebo as one capsule daily with breakfast. There was no formal restriction on randomization related to compliance during the run-in period. Randomization was carried out using a dynamic allocation method [17] with stratification for important prognostic factors, including age, sex, prior MI, lipid levels and urinary albumin excretion. All patients are being followed up through regular clinic visits to a clinic set in place for the purposes of the study as well as by routine health care provided by a regular diabetes clinic or specialist.

**Figure 1:**
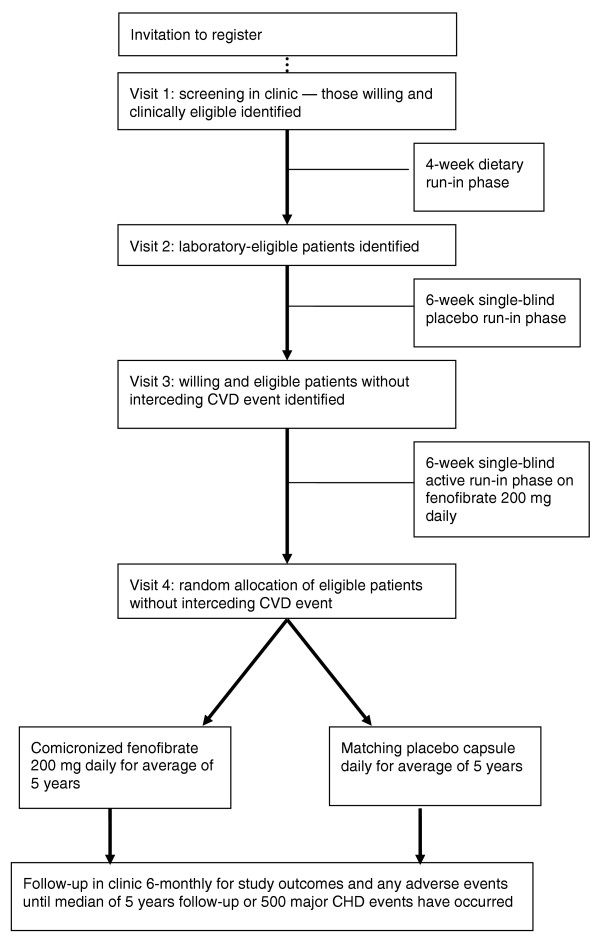
Study flow schema. CVD = cardiovascular disease.

The run-in phase for the study consists of a 4-week diet-only period, followed by a 6-week single-blind placebo period, then a 6-week single-blind active run-in period on comicronized fenofibrate 200 mg once daily for all patients, before randomization (Figure 1). This was to allow patients time to discuss long-term participation with their families and their usual doctors and for evaluation of the benefits of fenofibrate treatment on a background of recommended dietary advice. Further, the active run-in period was to determine to what extent any long-term clinical benefits of treatment correlate with the short-term effects of the drug to modify different lipid fractions.

Follow-up in the study will be for not less than 5 years of median duration and until a total of at least 500 first coronary events have accumulated in the trial, unless the study is terminated earlier by advice from the Safety and Data Monitoring Committee.

### Study outcomes

The principal study outcome is the combined incidence of first nonfatal MI or CHD death among all randomized patients during the scheduled treatment period (Table 2). Secondary outcomes include the effects of comicronized fenofibrate on major cardiovascular events (CHD events, total stroke and other cardiovascular death combined), total cardiovascular events (major cardiovascular events plus coronary and carotid revascularization), CHD death, total cardiovascular deaths, haemorrhagic and nonhaemorrhagic stroke, coronary and peripheral revascularization procedures, cause-specific non-CHD mortality (including cancer, suicide), and total mortality. All deaths, possible MIs and possible strokes are adjudicated in blinded fashion by the Outcomes Assessment Committee.

**Table 2:** Definitions for primary outcome assessment in the FIELD study

**Myocardial infarction**
Definite myocardial infarction = criterion 1; or any two of criteria 2 to 4; or criterion 5
1. New Q waves: new pathological Q waves (or Q-S pattern) of at least 0.03 seconds in width in at least 2 leads in the same lead group (in the absence of left bundle branch block)
2. Evolutionary ST-T wave changes: evolution of an injury current lasting more than one day and present in at least 2 leads in the same lead group: for example, ST elevation of 2 mm or more in anterior leads, *or *1 mm or more in inferior leads followed by T-wave inversion of 1 mm or more; this requires a minimum of two traces taken at least one day apart
3. Ischemic pain: history of typical ischemic pain lasting for at least 15 minutes *and *unresponsive to sublingual nitrates (if given)
4. Biochemical markers: elevation of CK or CKMB enzymes to over twice the upper limit of normal (for the laboratory) after the attack *or *elevation of troponin T to more than 0.1 μg/L or troponin I to levels above the upper limit of normal (for the laboratory)
5. Postmortem diagnosis: autopsy showing evidence of acute myocardial infarction.
**Death**
Coronary heart disease death = any of 1.1 to 1.7
1. Coronary
1.1 Definite fatal myocardial infarction: death following definite acute myocardial infarction in the preceding 28 days (and without an unrelated noncoronary cause of death), or autopsy-proven recent acute myocardial infarction
1.2 Sudden cardiac death: death occurring within one hour of onset of new cardiac symptoms or unwitnessed death after last having been seen without new symptoms; in each case, without any noncoronary disease that could have been rapidly fatal and without having been confined to hospital or other institution because of illness within 24 hours of death
1.3 Possible myocardial infarction: death in hospital with possible myocardial infarction (that is, typical ischaemic pain and ECG and enzyme results do not fulfil the criteria for definite myocardial and there is no good evidence for another event)
1.4 Resuscitated sudden death: documented cardiac arrest (in or out of hospital), after being resuscitated from what would have been sudden death; patient lives for more than one hour (hours to weeks).
1.5 Heart failure: death due to heart failure (prior grade 3–4 dyspnoea, NYHA) without any defined noncoronary cause
1.6 Death after coronary revascularisation: death (in the same admission) after any coronary revascularisation procedure (CABG or PTCA).
1.7 Other coronary: death where the underlying cause is certified as coronary (and there is no evidence for a noncoronary cause of death, clinically or at autopsy)
2. Noncoronary cardiac: death for which the underlying cause is certified as noncoronary cardiac disease
3. Vascular (noncardiac): death which is certified as vascular but not coronary disease: for example, cerebrovascular accident, pulmonary embolism, complications of peripheral vascular disease or uncontrolled hypertension
4. Cancer: death for which the underlying cause is certified as malignant neoplasm
5. Trauma: death where the underlying cause is certified as a wound or injury either accidental or inflicted
6. Suicide: death for which the underlying cause is certified as deliberate and voluntary taking of one's own life
7. Other: other cause of death not specified above.

Tertiary outcomes include the effects of treatment on development of vascular and neuropathic amputations, nonfatal cancers, the progression of renal disease, laser treatment for diabetic retinopathy, hospitalization for angina pectoris, and numbers and duration of all hospital admissions. The effects of treatment on the outcome of total cardiovascular events will be examined inThe rates of events various subgroups of particular interest, such as men and women, those <65 years and ≥ 65 years of age, by subgroup of each of baseline total cholesterol, HDL cholesterol, triglyceride and fibrinogen, baseline insulin use, or not, and the presence, or absence, at baseline of microalbuminuria.

The primary analysis will be of time to first study outcome, using standard log-rank methods [18,19], and where appropriate, proportional-hazards models with adjustment for covariates. Intention-to-treat methods, comparing all those allocated to comicronized fenofibrate with all those allocated to placebo, will be used.

### Sample size

The rates of events used for the original study power calculations were based on information from a variety of sources. During recruitment, when the numbers of participants with prior MI was falling well short of the number originally planned (in about 2000), the sample size was extended from the original total of 8000 to a final number of 9795 reached in November 2000. In late 2002, the statistical power of the trial was reviewed again. These reviews were planned in the original protocol design and were undertaken by reviewers completely blinded to all treatment allocation. The reassessment included information on final sample size, overall rate of discontinuation of study medication and commencement of open-label cholesterol treatment, and overall event rates in relation to CHD death, MI, and stroke.

After the review it was clear that the trial would yield too few deaths from CHD to retain sufficient power, over its planned duration of around 5 years, to show a significant reduction in this endpoint. The FIELD Management Committee determined that the primary outcome of the trial should be amended from CHD death to CHD events (that is, CHD death plus nonfatal MI, a decision made in December 2002). It was also decided to change the principal outcome for subgroup analyses to look at the effects of fenofibrate in subjects with and without prior cardiovascular disease (CVD) (originally those with and without prior MI).

For a primary outcome of CHD events (CHD death plus nonfatal MI), it is projected that approximately 500 CHD events will have occurred when 5 years median follow-up has elapsed (during the first quarter of 2005); by this time the trial will have 80% power to detect an observed 22% reduction in CHD events (based on the intention-to-treat method of analysis). This will also provide 90% power to detect a 25% relative reduction in CHD events (based on intention-to-treat analysis). Both calculations allow for the effects of an average drop-out rate from active treatment over the course of the study of 10% and a similar drop-in rate of 10% from placebo to open cholesterol-lowering therapy (Table 3). These allowances for loss of compliance require an increase in sample size of approximately 60% when compared with a study with no drop-outs from, or drop-ins to, active treatment.

**Table 3:** Predicted numbers of events and corresponding power in the FIELD study among 9795 people with diabetes, based on a median follow-up of 5 years

		**Allocated treatment**		Power to detect effect at 2*P *< 0.05*
**Risk category**		**Fenofibrate**	**Placebo**	
**Primary prevention (7683 with no prior CVD)**				
Total CVD events†		298	385	93%
**Secondary prevention (2112 with prior CVD)**				
Total CVD events		229	288	83%
**Men (6139)**				
Total CVD events		392	503	98%
**Women (3656)**				
Total CVD events		133	171	60%
**All patients (9795)**				
Total CVD events		525	675	99%
Total CHD events		219	281	80%

* Calculations assume a reduction in risk for each endpoint of around 27% with full compliance, resulting in an observed risk reduction of approximately 22% on intention-to-treat analysis; the risk in the prior CVD group is 2.75 times that for patients with no prior CVD, the risk in men is 1.75 times that in women.

† Expanded endpoint 'total CVD events' comprises cardiovascular death, nonfatal myocardial infarction, coronary artery bypass grafting, percutaneous transluminal coronary angioplasty, stroke and carotid revascularization.

CVD = cardiovascular, CHD = coronary heart disease

If the uptake of cholesterol-lowering therapy proves to be greater in the placebo group than in the fenofibrate-allocated group, the observed treatment effect of fenofibrate will underestimate its true efficacy.

### Safety and event monitoring

The trial has an independent Safety and Data Monitoring Committee to safeguard the patients' interests and to formally evaluate from time to time on a regular basis whether, for any reason, they would recommend that the study should be modified or stopped. Up to 5 formal interim analyses are planned, at time points to be determined by the Safety and Data Monitoring Committee, with a stringent nominal significance level (3 standard deviations; 2*P *= 0.003) to preserve an overall type 1 error probability of no more than 0.05. The events to be used for these analyses are counts of death from CHD. The Management Committee, the collaborators, the study sponsor and all the central administrative staff, with the exception of the unblinded statistician, will remain ignorant of the interim results for mortality and major morbidity. During the study, the group effects of treatment on biochemical parameters, such as lipid fractions, and other surrogate endpoints may be published, subject to prior approval of the Management Committee, provided that individual patient treatment assignments are not revealed. Patients are being monitored regularly by lipid profiles, liver function tests, creatine phosphokinase, fasting glucose, HbA1c, and urinary microalbumin. The study has been approved by local ethics committees at each participating institution, which also approved the information discussed and informed-consent procedures. The first patient in FIELD was registered in November 1997 and randomized in February 1998. The study has recruited 9795 patients; the final patient was randomized on 3 November 2000.

### Study sponsorship and organisation

The main sponsor of the trial and supplier of the fenofibrate and matching placebo medication, is Laboratoires Fournier S.A., Dijon, France. The study is also supported by the National Health and Medical Research Council of Australia through Unit, Program and Fellowship grants to the NHMRC Clinical Trials Centre. The study is being coordinated independently of the sponsors by the NHMRC Clinical Trials Centre, University of Sydney, Sydney, Australia and overseen by the study Management Committee. The study has been endorsed by the National Heart Foundation of Australia, Diabetes Australia, the New Zealand Society for the Study of Diabetes, and the Finnish Diabetes Association.

## Conclusion

In 1997, before the FIELD study commenced, the role of lipid modification in diabetes remained uncertain, except possibly for hypercholesterolaemic people with a prior MI. Two large-scale trials, the Scandinavian Simvastatin Survival Study (4S) [20] and the Cholesterol and Recurrent Events (CARE) [21] study, had showed that the use of 3-hydroxy-3-methylglutaryl-coenzyme A (HMG-CoA) reductase inhibitors, simvastatin and pravastatin, respectively, substantially reduced cardiovascular events hypercholesterolaemic and in general post-MI populations. But neither study included sufficient numbers of patients with diabetes (*n *= 202 and *n *= 586, respectively) to have the power to show reliably whether these benefits would translate into reductions in CHD mortality in the setting of diabetes, nor the effects in them of treatment on noncoronary events and mortality. Further, the West of Scotland (WOSCOPS) study of pravastatin in hypercholesterolaemic men with no prior CHD, which reported a marginally significant reduction in overall mortality, had fewer than 100 subjects with diabetes [22].

Since that time, numerous other trials of statin treatment have been reported, with randomized data now reported on over 18000 persons with diabetes. Those involving more than 1000 people with diabetes include the Long-Term Intervention with Pravastatin in Ischaemic Disease (LIPID) study [6,23], the Heart Protection Study [24,25], the Anglo-Scandinavian Cardiac Outcomes Trial (ASCOT) [26] and Antihypertensive and Lipid-Lowering Treatment to Prevent Heart Attack Trial (ALLHAT-LLT) [27]. Another trial, the Collaborative Atorvastatin Diabetes Study (CARDS), has stopped early, after about 4 years of follow-up, with results showing clear benefits of reduced cardiac and stroke events of using atorvastatin among 2838 people with diabetes and high cardiovascular risk [28]. Important new results have been communicated to investigators and patients so that it can be considered whether, during the follow-up of FIELD, statin therapy is now indicated for any individual. The protocol allows for statin therapy to be added at any time after randomization and recommends continuing study medication; thus the study is evaluating the role of fenofibrate on a background of usual care. This feature of the study design will contribute to the evidence about the safety of combined statin and fibrate therapy.

Two large-scale trials of fibrate therapy have also been completed: the Veterans Low-HDL Cholesterol Intervention Trial (VA-HIT) [29,30] and the Bezafibrate Infarct Prevention (BIP) [31] trial. Both studies were limited to people with prior MI and have reported reductions in major cardiovascular events among participants with low HDL and high TG at baseline, which were greater than those seen with use of the same fibrate among those without dyslipidaemia. The VA-HIT trial also reported reduced CHD mortality in those with diabetes receiving gemfibrozil and a reduced rate of cardiovascular events, although rates of nonfatal MI did not change significantly [32]. A third trial, the Diabetes Atherosclerosis Intervention Study (DAIS), showed reduced progression of established coronary atherosclerosis among those randomized to fenofibrate compared with those receiving matching placebo, over 3 years [33].

At the same time, our understanding of the mechanism of action of fibrates has grown, with identification of the peroxisome proliferator-activated receptor alpha (PPAR-alpha) transcription factor as the primary pathway through which fibrate-mediated effects are triggered [34,35]. The abundance of desirable effects of PPAR-alpha activation by fibrates has generated extraordinary interest in their role in the prevention of atherosclerosis via regulation of lipid metabolism, vascular inflammation, and haemostatic factors. The importance of changes in apolipoprotein B and non-HDL-cholesterol levels appears greater with fibrate therapy than with statin use [36], particularly in patients with type 2 diabetes [37]. Increased interest in the FIELD study has resulted, as it will generate clinical data on similar numbers of persons with diabetes to that available for the statins (Table 4) and will enlarge the range of lipid profiles studied and the number of events in such populations (Table 5).

**Table 4:** Unconfounded randomized controlled trials of lipid-lowering therapy, showing numbers of subjects with diabetes

**Study**	**Population**	**Year of primary publication**	**Therapy**	**Total no.**	**No. with diabetes**	**Reference**
4S	Prior CHD	1994	Simvastatin 20–40 mg	4444	202	20, 38
CARE	Prior CHD	1996	Pravastatin 40 mg	4159	586	21
Post-CABG*	Prior CHD	1997	Lovastatin 40–80 mg vs 2.5–5 mg	1351	122	39, 40
LIPID	Prior CHD	1998	Pravastatin 40 mg	9014	1077	6, 23
GISSI-P*	Prior CHD	2000	Pravastatin 20 mg	4271	582	41
GREACE*	Prior CHD	2002	Atorvastatin 10–80 mg	1600	313	42
PROSPER	Mixed	2002	Pravastatin 40 mg	5804	623	43
ALLHAT-LLT*	Mixed	2002	Pravastatin 20–40 mg	10355	3638	27
HPS	Mixed	2003	Simvastatin 40 mg	20536	5963	24, 25
ASCOT-LLA	Mixed	2003	Atorvastatin 10 mg	10305	2532	26
WOSCOPS	Primary	1995	Pravastatin 40 mg	6595	76	22
AFCAPS/TexCAPS	Primary	1998	Lovastatin 20–40 mg	6605	1 55	44
CARDS	Primary	2004	Atorvastatin 10 mg	2838	2838	28
**Total**	**–**	**–**	**Any statin**	**87877**	**18707**	
VA-HIT	Prior CHD	1999	Gemfibrozil 1200 mg	2531	769	29, 30, 32
BIP	Prior CHD	2000	Bezafibrate 400 mg	3090	309	31
DAIS	Mixed	2001	Fenofibrate 200 mg	418	418	33
LEADER	Mixed	2002	Bezafibrate 400 mg	1568	268	45
SENDCAP	Primary	1998	Bezafibrate 400 mg	164	164	46
HHS	Primary	1992	Gemfibrozil 1200 mg	4081	135	14, 15
**Total**	**–**	**–**	**Any fibrate**	**11852**	**2063**	

* No placebo used; lipid lowering compared with less treatment in Post-CABG study, with no treatment in GISSI-P study, and with usual care in ALLHAT-LLT and GREACE studies.

CHD = coronary heart disease

**Table 5:** Entry criteria and outcomes in trials of fibrates

**Study**	**Demographic entry criteria**	**Lipid entry criteria**	**Outcomes**
SENDCAP	men and women, 35–65 years, no cardiovascular disease	• serum cholesterol ≥ 5.2 mmol/L • triglyceride ≥ 1.8 mmol/L • HDL ≤ 1.1 mmol/L • total/HDL ≥ 4.7	change in carotid intima-media thickness, lipid changes, CHD events, at 3 years
VA-HIT	men, <74 years, documented history of CHD	• HDL ≤ 1.0 mmol/L • LDL ≤ 3.6 mmol/L • triglyceride ≤ 3.4 mmol/L	nonfatal MI or CHD death over median 5.1 years
BIP	men and women, 45–74 years, MI 6 months to 5 years before, no insulin-dependent diabetes	• serum cholesterol 4.7-6.5 mmol/L • LDL ≤ 4.7 mmol/L • HDL ≤ 1.2 mmol/L • triglyceride ≤ 3.4 mmol/L	fatal or nonfatal MI or sudden death over mean 6.2 years
DAIS	men and women, 40–65 years, type 2 diabetes	• total/HDL ≥ 4 • LDL 3.5–4.5 mmol/L + triglyceride ≤ 5.2 mol/L *or *LDL ≤ 4.5 mmol/L + triglyceride 1.7–5.2 mol/L	change in coronary artery lumen diameters, by angiography, and lipid changes after 3 years
LEADER	men with lower-extremity arterial disease, no lipid-lowering drug	• serum cholesterol 3.5–8.0 mmol/L	CHD events and stroke over median 4.6 years
HHS	men, 40–55 years, asymptomatic	• non-HDL cholesterol >5.2 mmol/L	lipid changes, MI, cardiac death at 5 years

CHD = coronary heart disease, MI = myocardial infarction, HDL = high-density lipoprotein, LDL = low-density lipoprotein

Approximately 140 million adults were estimated to be suffering from diabetes mellitus, the most common endocrine disorder worldwide, in 1997. By 2010, projections put diabetes prevalence about 60 percent higher, at 221 million. Just as many persons again have an elevated fasting glucose level, or impaired fasting glucose, which can progress rapidly to diabetes. Without the FIELD study, doctors would remain uncertain about the merits of using a fibrate when confronted with a patient with diabetes at risk of clinical CHD. It is expected that the main results of FIELD will be reported in late 2005.

## Declaration of competing interests

Of the Management Committee of the FIELD Study:

PB, YAK and RS have received reimbursements, fees, funding, or salary in the past five years from an organization that may in any way gain or lose financially from the publication of this paper;

No authors hold or have held stocks or shares in such an organization;

No authors have other financial competing interests;

AK has the following nonfinancial competing interests: Advisory board membership.

## Authors' contributions

The FIELD Management Committee conceived and developed the study protocol and are the responsible authors of this manuscript.

## Appendix 1: Study organization

### Management Committee

P Barter*, J Best*, P Colman, M d’Emden, T Davis, P Drury, C Ehnholm, P Glasziou, D Hunt, A Keech* (study chairman and principal investigator), YA Kesaniemi, M Laakso, R Scott*, RJ Simes*, D Sullivan, M-R Taskinen*, M Whiting; J-C Ansquer, B Fraitag (non-voting sponsor representatives). * Executive Committee members.

### Outcomes Assessment Committee

N Anderson, G Hankey, D Hunt (chairman), S Lehto, S Mann, M Romo; LP Li (outcomes officer, in attendance).

### Safety and Data Monitoring Committee

C Hennekens, S MacMahon (chairman), S Pocock, A Tonkin, L Wilhelmsen; P Forder (unblinded statistician, in attendance). 

### Site principal investigators

***Australia***: H Akauola, F Alford, P Barter, I Beinart, J Best, S Bohra, S Boyages, P Colman, H Connor, D Darnell, T Davis, P Davoren, F Lepre, F De Looze, M d'Emden, A Duffield, R Fassett, J Flack, G Fulcher, S Grant, S Hamwood, D Harmelin, R Jackson, W Jeffries, M Kamp, L Kritharides, L Mahar, V McCann, D McIntyre, R Moses, H Newnham, G Nicholson, R O'Brien, K Park, N Petrovsky, P Phillips, G Pinn, D Simmons, K Stanton, B Stuckey, D R Sullivan, M Suranyi, M Suthers, Y Tan, M Templer, D Topliss, J H Waites, G Watts, T Welborn, R Wyndham; ***Finland: *** H Haapamaki, A Kesaniemi, M Laakso, J Lahtela, H Levanen, J Saltevo, H Sodervik, M Taskinen, M Vanhala; ***New Zealand: ***J Baker, A Burton, P Dixon, J Doran, P Drury, P Dunn, N Graham, A Hamer, J Hedley, J Lloyd, P Manning, I McPherson, S Morris, C Renner, R Scott, R Smith, M Wackrow, S Young.

### Co-investigators and site coordinators

***Australia: ***F Alard, J Alcoe, F Alford, C Allan, J Amerena, R Anderson, N Arnold, T Arsov, D Ashby, C Atkinson, L Badhni, M Balme, D Barton, B Batrouney, C Beare, T Beattie, J Beggs, C Bendall, C Bendall, A Benz, A Bond, R Bradfield, J Bradshaw, S Brearley, D Bruce, J Burgess, J Butler, M Callary, J Campbell, K Chambers, J Chow, S Chow, K Ciszek, P Clifton, P Clifton-Bligh, V Clowes, P Coates, C Cocks, S Cole, D Colquhoun, M Correcha, B Costa, S Coverdale, M Croft, J Crowe, S Dal Sasso, W Davis, J Dunn, S Edwards, R Elder, S El-Kaissi, L Emery, M England, O Farouque, M Fernandez, B Fitzpatrick, N Francis, P Freeman, A Fuller, D Gale, V Gaylard, C Gillzan, C Glatthaar, J Goddard, V Grange, T Greenaway, J Griffin, A Grogan, S Guha, J Gustafson, P S Hamblin, T Hannay, C Hardie, A Harper, G Hartl, A Harvey, S Havlin, K Haworth, P Hay, L Hay, B Heenan, R Hesketh, A Heyworth, M Hines, G Hockings, A Hodge, L Hoffman, L Hoskin, M Howells, D Hunt, A Hunt, W Inder, W Inder, D Jackson, A Jovanovska, K Kearins, P Kee, J Keen, D Kilpatrick, J Kindellan, M Kingston-Ray, M Kotowicz, A Lassig, M Layton, S Lean, E Lim, F Long, L Lucas, D Ludeman, D Ludeman, C Ludeman-Robertson, M Lyall, L Lynch, C Maddison, B Malkus, A Marangou, F Margrie, K Matthiesson, J Matthiesson, S Maxwell, K McCarthy, A McElduff, H McKee, J McKenzie, K McLachan, P McNair, M Meischke, A Merkel, C Miller, B Morrison, A Morton, W Mossman, A Mowat, J Muecke, P Murie, S Murray, P Nadorp, S Nair, J Nairn, A Nankervis, K Narayan, N Nattrass, J Ngui, S Nicholls, V Nicholls, JA Nye, E Nye, D O'Neal, M O'Neill, S O'Rourke, J Pearse, C Pearson, J Phillips, L Pittis, D Playford, L Porter, L Porter, R Portley, M Powell, C Preston, S Pringle, W A Quinn, J Raffaele, G Ramnath, J Ramsden, D Richtsteiger, S Roffe, S Rosen, G Ross, Z Ross, J Rowe, D Rumble, S Ryan, J Sansom, C Seymour, E Shanahan, S Shelly, J Shepherd, G Sherman, R Siddall, D Silva, S Simmons, R Simpson, A Sinha, R Slobodniuk, M Smith, P Smith, S Smith, V Smith-Orr, J Snow, L Socha, T Stack, K Steed, K Steele, J Stephensen, P Stevens, G Stewart, R Stewart, C Strakosch, M Sullivan, S Sunder, J Sunderland, E Tapp, J Taylor, D Thorn, D Thorn, A Tolley, D Torpy, G Truran, F Turner, J Turner, J van de Velde, S Varley, J Wallace, J Walsh, J Walsh, J Walshe, G Ward, B Watson, J Watson, A Webb, F Werner, E White, A Whitehouse, N Whitehouse, S Wigg, J Wilkinson, E Wilmshurst, D Wilson, G Wittert, B Wong, M Wong, S Worboys, S Wright, S Wu, J Yarker, M Yeo, K Young, J Youssef, R Yuen, H Zeimer, R W Ziffer;  ***Finland: *** A Aura, A Friman, J Hanninen, J Henell, N Hyvarinen, M Ikonen, A Itkonen, J Jappinen, A Jarva, T Jerkkola, V Jokinen, J Juutilainen, H Kahkonen, T Kangas, M Karttunen, P Kauranen, S Kortelainen, H Koukkunen, L Kumpulainen, T Laitinen, M Laitinen, S Lehto, R Lehto, E Leinonen, M Lindstron-Karjalainen, A Lumiaho, J Makela, K Makinen, L Mannermaa, T Mard, J Miettinen, V Naatti, S Paavola, N Parssinen, J Ripatti, S Ruotsalainen, A Salo, M Siiskonen, A Soppela, J Starck, I Suonranta, L Ukkola, K Valli, J Virolainen; ***New Zealand: ***P Allan, W Arnold, W Bagg, K Balfour, T Ball, B Ballantine, C Ballantyne, C Barker, C Barker, F Bartley, E Berry, G Braatvedt, A Campbell, T Clarke, R Clarke, A Claydon, S Clayton, P Cresswell, R Cutfield, J Daffurn, J Delahunt, A Dissnayake, C Eagleton, C Ferguson, C Florkowski, D Fry, P Giles, M Gluyas, C Grant, P Guile, M Guolo, P Hale, M Hammond, M Hammond, P Healy, M Hills, J Hinge, J Holland, B Hyne, A Ireland, A Johnstone, S Jones, G Kerr, K Kerr, M Khant, J Krebs, L Law, B Lydon, K MacAuley, R McEwan, P McGregor, B McLaren, L McLeod, J Medforth, R Miskimmin, J Moffat, M Pickup, C Prentice, M Rahman, E Reda, C Ross, A Ryalls, D Schmid, N Shergill, A Snaddon, H Snell, L Stevens, A Waterman, V Watts.

### Coordinating centre teams

***NHMRC Clinical Trials Centre, Sydney: ***K Jayne, E Keirnan, P Newman, G Ritchie, A Rosenfeld (project directors), E Beller, P Forder, V Gebski, A Pillai (study statisticians), C Anderson, S Blakesmith, S-Y Chan, S Czyniewski, A Dobbie, S Doshi, A Dupuy, S Eckermann, M Edwards, N Fields, K Flood, S Ford, C French, S Gillies, C Greig, M Groshens, J Gu, Y Guo, W Hague, S Healy, L Hones, Z Hossain, M Howlett, J Lee, L-P Li, T Matthews, J Micallef, A Martin, I Minns, A Nguyen, F Papuni, A Patel, J Pearse, R Pike, M Pena, K Pinto, D Schipp, J Schroeder, B Sim, C Sodhi, T Sourjina, C Sutton, R Taylor, P Vlagsma, S Walder, R Walker, W Wong, J Zhang, B Zhong, A Keech (deputy director), RJ Simes (director); ***Helsinki Project Office: ***A Kokkonen, P Narva, E-L Niemi, A Salo, A-M Syrjanen, M-R Taskinen (director); ***Christchurch Project Office: ***C Lintott, R Scott (director).

### Central laboratories

***Adelaide: ***R Tirimacco, M Whiting; ***Helsinki: ***C Ehnholm, M Ikonen, M Kajosaari, L Raman, J Sundvall, M Tukianen. .

**Laboratoires Fournier SA liaison: ***Dijon: *J-C Ansquer, B Fraitag, D Crimet, I Sirugue*Sydney: *P Aubonnet.
